# A case report about anatomy applications for a physical therapy hybrid online curriculum

**DOI:** 10.5195/jmla.2020.825

**Published:** 2020-04-01

**Authors:** Kathryn L. Havens, Nicole A. Saulovich, Karin J. Saric

**Affiliations:** Division of Biokinesiology and Physical Therapy, University of Southern California, Los Angeles, CA, khavens@usc.edu, https://orcid.org/0000-0002-9865-9879; Division of Biokinesiology and Physical Therapy, University of Southern California, Los Angeles, CA, saulovic@usc.edu; Norris Medical Library, University of Southern California, Los Angeles, CA, ksaric@usc.edu

## Abstract

**Background:**

Three-dimensional digital anatomy applications can provide a powerful supplement to more traditional learning modalities. The challenge for medical libraries and educators is to select an app that best supports anatomical learning objectives and then effectively integrate it into health sciences curricula. App selection is particularly important when traditional learning modalities, such as cadaver dissection, are not feasible. Selection was a challenge at the authors’ university, as the doctor of physical therapy (DPT) program expanded into a hybrid online environment.

**Case Presentation:**

Reported here are our: (1) analysis and identification of an anatomy app to supplement cadaver lab instruction for DPT students who were enrolled in a hybrid program, where the majority of instruction took place online; (2) description of the implementation process; and (3) discussion of student feedback and the library’s perspective. Features and shortcomings of two anatomy apps, Complete Anatomy (CA) 2019 by 3D4 Medical and Human Anatomy Atlas (HAA) 2019 by Visible Body, were reviewed. CA was selected based on smoother navigation, visually appealing graphics, and user customization tools. The library purchased 1,000 CA redemption codes as a pilot program. Video recordings and live demonstrations of the app were used for instruction. Student feedback indicated extensive use. Based on success of the pilot, the library will purchase additional licenses.

**Conclusions:**

Medical libraries can use our experience as an example to help select anatomy resources that would be useful when considering the conversion of health sciences programs into online environments and further guide app integration to supplement other anatomical models.

## BACKGROUND

Medical libraries provide essential resources for anatomy students. Beyond traditional textbooks, library licensing can also allow students to use digital atlases to supplement their anatomy education [1, 2] with three-dimensional (3D) applications that provide a virtual lab experience with an interactive atlas. The challenge, both for medical librarians and for anatomy instructors, is to select and effectively implement a 3D anatomy app, particularly for health sciences curricula that depend heavily on knowledge of anatomy, such as physical therapy (PT).

A thorough understanding of 3D musculoskeletal anatomy is essential for PT practitioners, as manual skills and treatment interventions rely on clear mental images of underlying structures. For understanding anatomy, a cadaver dissection has historically been considered a sacrosanct experience [2]. This traditional teaching modality provides a valuable understanding of the intricacies of human tissue, 3D nature of the body, and important relationships between neurovascular and muscular structures. However, cadaver programs are extremely expensive, and dissection takes hours to perform and prolongs exposure to caustic chemicals. Thus, anatomy education has evolved to include alternative strategies, such as plastic models and digital atlases.

While some studies conclude that certain aspects of a dissection lab are irreproducible, overall findings concur that anatomy apps can play a powerful role as supplemental instructional resources [1–5]. The technology also affords PT students the possibility to engage with content beyond what is feasible in a traditional cadaver lab, including the ability to view micro-detail of anatomical structures, joint movement, muscle attachments and actions, and neurovascular pathways. Digital apps are particularly useful when transitioning a curriculum from residential experiences to hybrid programs that include a combination of online and on-campus experiences [6]. Implementation of apps into the curricular redesign allows instructors to extract the value of traditional instructional modalities and supplement this information for online environments [1–5]. Given their value, the challenge for educators becomes selecting the anatomy app that best fits students’ needs and includes features that support optimal instruction.

In 2017, the University of Southern California Division of Biokinesiology and Physical Therapy decided to create a hybrid doctor of physical therapy program to mirror the on-campus residential program. As anatomy faculty began translating course content to an online platform, the authors reached out to and partnered with the Norris Medical Library staff to identify appropriate resources for the program.

## CASE PRESENTATION

Although the medical library already licensed the Human Anatomy Atlas (HAA) by Visible Body, the app did not appear robust enough to support the conversion of anatomy instruction into an online platform. Some features lacking from HAA included high-resolution, micro-detailed models, sophisticated mark-up tools, and ease of navigation. Though there are many available products, we decided to focus on one other commonly used anatomy app, Complete Anatomy (CA) by 3D4Medical. We based this choice on a literature search [1, 3, 5, 7], its number one ranking on the Apple App Store for medical apps, and informal questioning of our own anatomy students’ app preferences.

To understand whether CA could better address student and instructor needs, we conducted a detailed comparison of the two apps. The most updated versions were evaluated using an iPad Pro: CA 2019 (version 4.0.4) [8] and HAA 2019 (version 2019.2.49) [9]. Apple iPads are required for our students. Assessed features included content and functionality, quality of and ability to manipulate models, user customization tools, and micro-detail of anatomical structures (supplemental Appendix A).

In this case report, we discuss the outcomes of our comparison, library licensing, and app implementation. In addition, we explore how our experiences extend the practice of health sciences librarianship and education.

### Resource comparison

#### Common features of both apps

**Figure 1:**
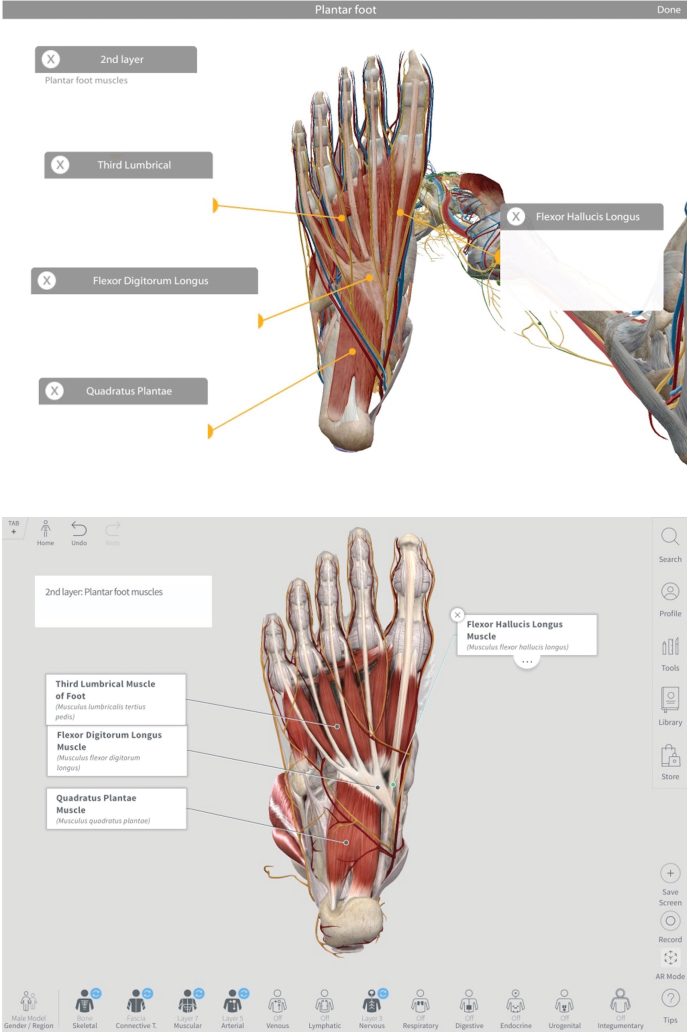
Select plantar foot anatomy Top: Human Anatomy Atlas, copyright 2018, Visible Body [9]; all rights reserved. Bottom: Complete Anatomy [8].

CA and HAA are both compatible with any Apple or Windows device and have similar intended audiences (supplemental Appendix A). Both apps provide the learner with a 3D interactive atlas (Figure 1) and include coverage of similar content (Table 1). They both offer a 360-degree experience of anatomy, including the ability to select any structure and then apply additional options of fading or hiding structures. Structure descriptions are provided, as well as associated clinical or medical conditions. Important for musculoskeletal anatomy, muscle attachments, blood supply, and innervation can be viewed. Bony landmarks can also be visualized and highlighted. Additionally, a high-tech augmented reality feature allows the user to overlay anatomy models onto any surface.

**Table 1:**
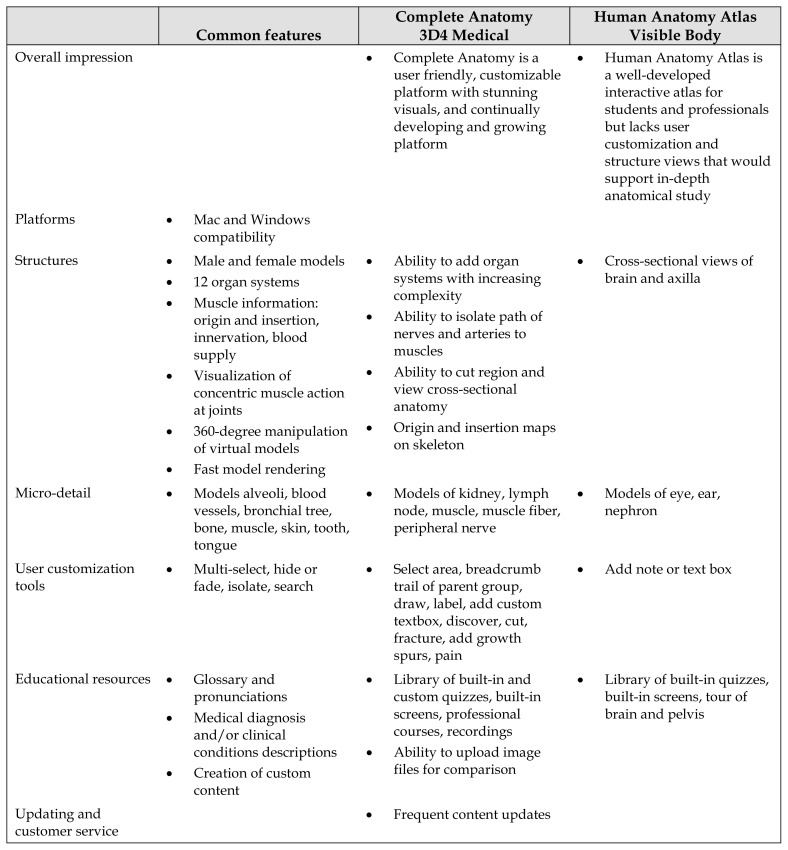
Resource comparison of Complete Anatomy (CA) and Human Anatomy Atlas (HAA) apps

	Common features	Complete Anatomy 3D4 Medical	Human Anatomy Atlas Visible Body
Overall impression		Complete Anatomy is a user friendly, customizable platform with stunning visuals, and continually developing and growing platform	Human Anatomy Atlas is a well-developed interactive atlas for students and professionals but lacks user customization and structure views that would support in-depth anatomical study
Platforms	Mac and Windows compatibility		
Structures	Male and female models 12 organ systems Muscle information: origin and insertion, innervation, blood supply Visualization of concentric muscle action at joints 360-degree manipulation of virtual models Fast model rendering	Ability to add organ systems with increasing complexity Ability to isolate path of nerves and arteries to muscles Ability to cut region and view cross-sectional anatomy Origin and insertion maps on skeleton	Cross-sectional views of brain and axilla
Micro-detail	Models alveoli, blood vessels, bronchial tree, bone, muscle, skin, tooth, tongue	Models of kidney, lymph node, muscle, muscle fiber, peripheral nerve	Models of eye, ear, nephron
User customization tools	Multi-select, hide or fade, isolate, search	Select area, breadcrumb trail of parent group, draw, label, add custom textbox, discover, cut, fracture, add growth spurs, pain	Add note or text box
Educational resources	Glossary and pronunciations Medical diagnosis and/or clinical conditions descriptions Creation of custom content	Library of built-in and custom quizzes, built-in screens, professional courses, recordings Ability to upload image files for comparison	Library of built-in quizzes, built-in screens, tour of brain and pelvis
Updating and customer service		Frequent content updates	
Areas for improvement	Unable to move joints for appropriate arthrokinematics Unable to view combined muscle action (e.g., hip extension with external rotation) Idealized representation of standard patient model	Superficial central nervous system anatomy	Moderate resolution of gross and microanatomy model graphics Yearly updates and quarterly bug fixes All or nothing addition of organ systems Less sophisticated tools Only select cross-sectional anatomy

Particularly important for PT education, individual concentric muscle actions can be isolated and viewed from different angles in both apps. Sophisticated animation shows the lengthening and shortening of the muscle with joint movement. This is critically important for PT students to visualize, as movement analysis is paramount in their education. However, we identified two shortcomings in both apps in relation to the needs of PT students. First is the ability to visualize arthrokinematic joint motion, which is movement at the articular surfaces of the bones and is the basis of manipulations in PT. Second, while both apps show individual joint motions, neither is capable of showing a customizable combined joint or functional movements, such as pronation and supination of the forearm when the elbow is flexed or combined motion of hip extension with external rotation.

#### Structure selection and isolation

Model interaction and manipulation is essential to an anatomy app’s utility. To customize atlas images, users need to efficiently navigate to a desired area and easily select or hide structures. Here, the two apps differ significantly. CA users can add increasingly complex layers of twelve organ systems to a selected body region, such as the full body or just the right upper arm. They can fade or hide single or multiple items. Clear visualization of nerve pathways, branching vasculature, and a breadcrumb trail to select parent structures all add to the user experience. By contrast, HAA presents screens in a main menu, and users need to know whether to navigate to regional, systemic, cross-sectional, micro-anatomy, or gross anatomy lab submenus. Specific body regions cannot be isolated; although structures can be added or removed, the full body model remains. In addition, the inability to gradually add organ systems makes building customized models difficult.

#### Micro-detail

**Figure 2:**
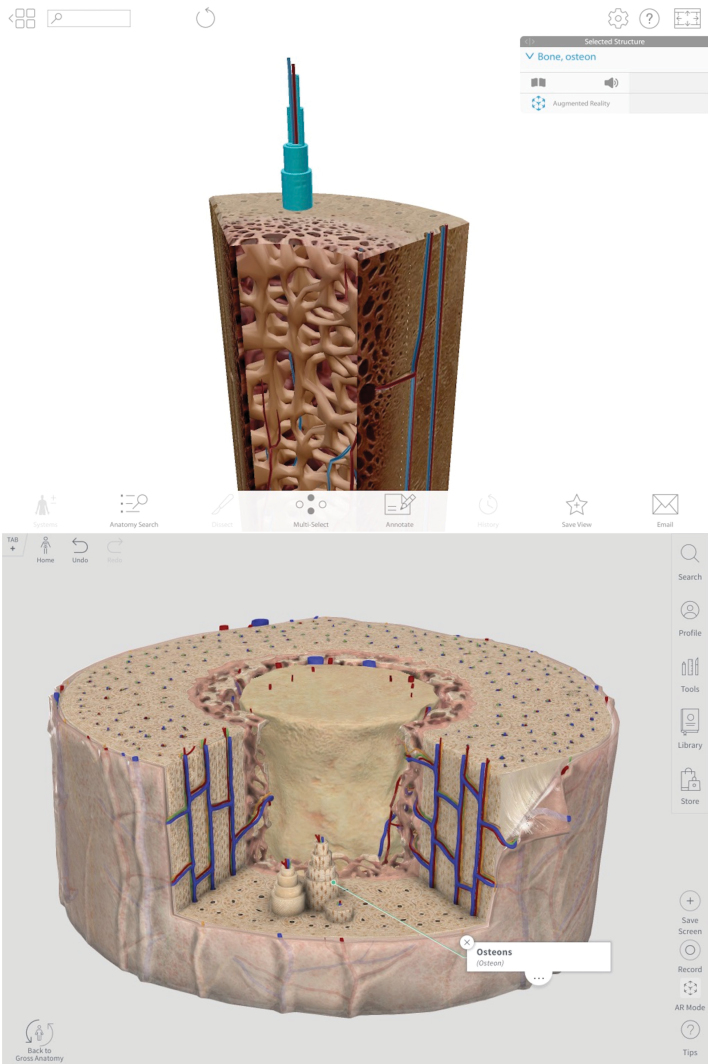
Micro-anatomy of bone cross-section Top: Human Anatomy Atlas, copyright 2018, Visible Body [9]; all rights reserved. Bottom: Complete Anatomy [8].

CA surpasses HAA in terms of micro-detail due to its higher resolution, particularly in close-up views. Accurate bony landmarks and clearer visualization of surfaces make users feel like they are manipulating a real bone. Bony parts, surfaces, landmarks, and red or blue origin or insertion maps that demonstrate the relationship between muscle attachments are all present. Additionally, microscopic models appear clearer in CA, with additional attention paid to detailed graphical representation of structures (Figure 2).

While the apps demonstrate the brain and central nervous system in gross anatomy, both leave something to be desired. HAA provides cross-sections of some brain areas and screens demonstrating special senses, which are not available in CA; however, resolution is low. Neither provides locations of important nuclei or pathways. Currently, neither contains the features necessary for neuroanatomy education.

#### User customization tools

Users can customize their atlas models through tools. While both apps allow users to label structures, add text boxes for custom notes, and use a 2D drawing tool, CA includes a variety of unique features that are not available in HAA. First, it allows users to attach a drawing or label on the model in 3D, allowing it to be viewed from any angle. To better understand pathologies, users can create growths or bone spurs, add pain or inflammation, or fracture bones. Users can “blow up” components to better understand spatial relationships. Finally, users can upload image files and view them next to the model, which helps address the issue of learning from an idealized standard model. It can also be useful for discussing content that benefits from comparison, such as differences from cadaver dissection or effects of different disease stages.

#### Educational resources

Both apps strive to support educators by including the ability to create customized content and quizzes. HAA’s courseware allows instructors to create custom quizzes and track students’ progress. However, CA’s content builder gives more flexibility. Instructors can create materials such as saved screens and custom quizzes and then record short lectures using these materials in the app. These can be shared publicly, or a single class can purchase access for an additional fee. Students can additionally view and buy video packs and expert-led courses from the 3D4Medical group that come as part of more advanced packages or are available with in-app purchasing. HAA’s courseware also allows instructors to create custom quizzes and track students’ progress, but custom model views cannot be shared directly with students.

#### Updating and customer service

CA has an academic review board that provides research and updates weekly. Their customer service team replies to complaints on purchasing platforms and discussion forums. HAA provides major software updates yearly and smaller bug fixes quarterly. For technical support, a dedicated contact person may be available through institutional relationships. However, for the general user, technical support through email can take an extended period of time.

### The authors’ choice: Complete Anatomy

Based on the results of testing detailed features in each app, we have found CA better able to address the specific needs of hybrid PT students who are studying anatomy in an online environment. While both apps provide the learner with many useful features, CA allows smoother navigation, contains visually appealing graphics, and provides unique tools that allow creative customization of content. These distinct features afford better tailoring of content to supplement instruction that is traditionally provided in cadaver labs.

As faculty members were able to demonstrate that CA contained distinct features that addressed their needs, the library agreed to pilot the resource based on 2 conditions: (1) the resource could be made available to faculty and students beyond the single division, and (2) individual licenses could be redeemed in an efficient and independent manner. An initial one-time purchase of 1,000 redemption codes for CA was made in summer 2018, and an online platform was created by the vendor to allow any USC faculty or student to independently redeem the codes. Redemption required a USC email address, and only 1 license was issued per person; however, users could download the app onto multiple devices. The online redemption platform allowed librarians to easily generate student awareness by embedding the access link on the library website and in guides that highlight key resources. Typically, students do not retain access to library-licensed resources upon graduation. However, the CA license allowed students to maintain continual access to the latest version that they owned upon graduation. Students who had already purchased the app were refunded by the vendor. Within 1 year, over 75% (n=773) of codes had been redeemed, out of a total estimated population of 11,931 students and faculty. At most, the PT division would have redeemed 200 codes, thus indicating broader campus usage.

### App integration

To encourage student interaction with the app, it was integrated into the first semester PT musculoskeletal anatomy course in several ways as a supplement to traditional lectures and cadaver lab experiences. First, to mitigate the software learning curve [1–3], the course began with a short video to describe how the app would be used to supplement learning and suggest tips for navigation. This familiarization process provided a baseline for students to independently navigate the app throughout the course.

Following a week’s worth of online lecture and lab content about each major joint of the body, the app was used to provide a short but formal review. Faculty led students through important structures of a specific joint by building numerous custom screens in the content builder that were quickly pulled up during demonstrations. Capturing the app screen with expert voice-over provided the students with additional opportunities to review the material and experience the 3D anatomy, while optimizing their own app skills. These lectures were recorded both in the app and in a professional video-recording studio.

Finally, faculty used CA to review structures during weekly live video sessions with students. For example, during the ankle and foot week, students spent time in small groups reviewing important ligaments’ attachment sites and functions. Next, faculty shared their app screens to review these ligaments and relevant bony landmarks. A major advantage of this screen sharing, rather than reviewing 2D text book images, was the ability to quickly add or remove layers of ligament and tendon and rotate the view to appreciate the structures’ 3-dimensionality. The purpose of these activities was to support anatomy education through visualizing and identifying 3D structures.

To gain insight into the student experience with CA, a post-semester informal student survey (supplemental Appendix B), with a 54% (25/46) response rate, was collected. The USC Health Sciences Institutional Review Board concluded that the survey did not qualify as human subjects’ research and was thus exempted from review. A majority (75%) indicated that they used the app at least once a week, and 20% used it every day. All respondents indicated that CA was useful to help them understand the 3D relationships between structures, with graphics and resolution of the model (68%) cited as the most useful feature. All respondents indicated that they were likely to use the app in future professional endeavors, and all but 1 indicated that they would recommend or had recommended it to someone outside of the program. Among aspects that students had difficulty with, navigation to structures and the software learning curve were most cited.

## DISCUSSION

The purpose of this case study was to describe one institution’s identification and implementation of an anatomy app to supplement cadaver lab instruction. While previous research has shown that anatomy apps have been successful in supplementing cadaver dissection [3, 5, 10–12], one study concluded that student perception of the value of apps has not always mirrored such findings [11]. Additionally, there still has been resistance among faculty to implement such resources into instruction: “Part of the reluctance may be related to the lack of an app database that includes a professional review of app content and accuracy” [3].

This case presentation addresses the current gap in literature by providing a detailed review of two of the most highly used anatomy apps [1, 3, 5, 7]. Our analysis demonstrates that CA provides a more complete view of anatomical micro-detail and understanding beyond dissection and serves as an exceptional supplemental resource for a hybrid PT program. CA also affords the possibility of personalizing education beyond the one semester anatomy course, as there is no time limit to student interaction with instructional material.

The student survey results are in alignment with previous research indicating extensive use of anatomy applications by chiropractic students [7] and studies that found positive perceptions of app effectiveness to support learning [12, 13]. Based on their feedback, we plan to update our course next year with more detailed guidance, emphasizing finding desired structures and creating custom 3D images. Faculty-led live sessions will instruct about using the app throughout the semester, and more assignments will be integrated to promote interaction with the app, reinforce its utility, and increase student confidence.

For the library, this experience demonstrated the need to align collection development plans with the shift we see in the communities we serve [14]: a shift to online instruction. Criteria for selecting mobile resources mirror those of physical items in many ways, in other words, content, cost, currency, scope, subject relevance, access, and updates [15]. However, additional factors must also be considered due to the digital format of the resource [14–16], including consideration of supported platforms; ease of authentication; technical feasibility, implementation, and compatibility with other library hardware and software; functionality and reliability; access options to easily disseminate licenses; promotion of the resource; persistency of content; and updates and vendor support [15–17]. In our case, we also had to consider discipline-specific needs, such as the app’s ability to replace information acquired during cadaver dissection and the duplication of resources because the library already licensed HAA.

While the pilot program was driven by the needs of one program, it demonstrated a clear need for CA across the USC Health Sciences Campus. In the upcoming year, the library will purchase additional codes and will promote this resource to other potential groups on the undergraduate campus. We were also able to identify a manner for easily disseminating individual licenses, which removed a barrier that prevented the purchase of app licenses in the past. We hope that other institutions can use this case study not only to inform selection and purchasing of anatomy apps, but also as a resource to expand best practices by including criteria for purchasing mobile resources in their collection development plans.

“To adequately plan for the future, it is important to understand how instructional practices and institutional pressures might change in the future” [2]. Expansion of the PT program into an online platform forced us to reimagine how we would provide our students with the necessary exposure to anatomy instruction. Although the anatomy culture has seen much resistance to education reform beyond the cadaver lab [4, 18, 19], appreciation for the value of apps as supplemental resources can change with time [3] and dissemination of research findings [18]. We hope that our successful implementation of this resource serves to reduce uncertainty around the adoption of apps to support anatomy learning [18].

While we agree with other authors that dissection provides unique and valuable educational experiences of real live anatomy that are unattainable by advanced anatomy apps, anatomy applications can serve as powerful supplements to gross anatomy education [20]. With the ever-evolving and increasing sophistication of anatomy apps, students will continue to benefit and obtain value from digital technology learning resources, as long as they are well implemented and supported by integrated instruction. Based on our comparison of the two most-used 3D digital anatomy apps, we have found the 3D4 Medical Complete Anatomy app to be a robust resource that is capable of supporting transition of a cadaver lab curriculum to an online instructional platform. As academic programs continue to develop distance learning programs, faculty can partner with librarians to identify and implement useful technologies to facilitate and supplement the conversion of residential programs into online platforms.

## SUPPLEMENTAL FILES

Appendix A Detailed comparison of features of Complete Anatomy and Human Anatomy Atlas

Appendix B Complete Anatomy app student feedback survey questions

## 

**Kathryn L. Havens**, khavens@usc.edu, https://orcid.org/0000-0002-9865-9879, Division of Biokinesiology and Physical Therapy, University of Southern California, Los Angeles, CA

**Nicole A. Saulovich**, saulovic@usc.edu, Division of Biokinesiology and Physical Therapy, University of Southern California, Los Angeles, CA

**Karin J. Saric**, ksaric@usc.edu, Norris Medical Library, University of Southern California, Los Angeles, CA
