# Supplementary material for: A case report about anatomy applications for a physical therapy hybrid online curriculum
Source: J Med Libr Assoc. 2020 Apr 1;108(2):295–303. doi: 10.5195/jmla.2020.825 (PMC7069813; doi:10.5195/jmla.2020.825)
Supplement: Appendix A [file jmla-108-295-s001.pdf]

JMLA Journal of the Medical Library Association

Supplemental content to
J Med Libr Assoc. Apr;108(2):dx.doi.org/10.5195/jmla.2020.825
www.jmla.mlanet.org
© Havens, Saulovich, Saric 2020

A case report about anatomy applications for a physical therapy hybrid online curriculum

Kathryn L. Havens; Nicole A. Saulovich; Karin J. Saric

APPENDIX A

Detailed comparison of features of Complete Anatomy and Human Anatomy Atlas

	Complete Anatomy<br>3D4 Medical	Human Anatomy Atlas<br>Visible Body
**Platforms**		
Compatibility	iPad, iPhone, Mac, Windows, and Android devices	iPad, iPhone, Mac, Windows, and Android devices
Operating system requirements	iPad/iPhone iOS 10.3, Mac OS 10.12, Windows 10, Android 7.0	iPad/iPhone iOS 11.3, Mac OS X 10.9.0, Windows 7/8/10
Cost	$44.99	$34.99
Site licensing	Yes	Yes
**Content**		
**Volumetric data**		
Data used to create models	Academic review board (professors and medical professionals) ensures accurate and relevant content; cites relevant literature in structure information	Models created by biomedical visualization experts and sent for review by outside team of professors and medical professionals
Accuracy and reliability of model	Highly accurate	Highly accurate
Update frequency	Weekly updates	Quarterly updates
Intended audience	Undergraduate, graduate students, health care professionals, academics for course instruction	Undergraduate, graduate students, health care professionals, academics for course instruction
**Quality of virtual models**		
Model sex	Male and female	Male and female
Number of anatomical systems	12: skeletal, connective tissue, muscular, arterial, venous, lymphatic, nervous, respiratory, digestive, endocrine, urogenital, integumentary	12: skeletal, connective tissue, muscular, arterial, venous, lymphatic, nervous, respiratory, digestive, endocrine, urogenital, integumentary
Comprehensiveness of anatomical structures	Excellent	Excellent

JMLA Journal of the Medical Library Association

SC
Supplemental content to
J Med Libr Assoc. Apr;108(2):dx.doi.org/10.5195/jmla.2020.825
www.jmla.mlanet.org
© Havens, Saulovich, Saric 2020

	**Complete Anatomy**<br>**3D4 Medical**	**Human Anatomy Atlas**<br>**Visible Body**
Number of layers	• skeletal 5<br>• connective tissue 7<br>• muscular 7<br>• arterial 5<br>• venous 3<br>• lymphatic 1<br>• nervous 3<br>• respiratory 1<br>• digestive 1<br>• endocrine 1<br>• urogenital 1<br>• integumentary 3	No layers: each system can only be added or removed
Cross-sectional anatomy	Customizable	Select brain and axial
Micro-detail	Exceptional detail	Moderate detail
**Musculoskeletal content**		
Muscle origin/insertion	Yes	Yes
Muscle innervation	Yes	Yes
Muscle blood supply	Yes	Yes
Muscle action animation	Yes	Yes
Isolate paths of nerves to muscle	Yes	No
Origin paths of arteries to muscle	Yes	No
**Navigation and tools**		
360-degree rotation	Yes	Yes
Number of view points	All	All
Rendering speed during manipulation	Fast	Fast
Ease of use of menu and learning curve	Moderate: many features extend customization process	Moderate: preset views reduce customization
Multi-select	Yes	Yes
Select area	Yes	No
Isolate, hide, fade	Yes	Yes

JMLA Journal of the Medical Library Association

SC
Supplemental content to
J Med Libr Assoc. Apr;108(2):dx.doi.org/10.5195/jmla.2020.825
www.jmla.mlanet.org
© Havens, Saulovich, Saric 2020

	**Complete Anatomy 3D4 Medical**	**Human Anatomy Atlas Visible Body**
Save screen	Yes	Yes
Labels	Yes: custom and built in	Yes
Search	Yes	Yes
Tools	Yes: text box, pen, 2D and 3D draw, cut, fractures, growth, spurs, pain, discover	Yes: text box, 2D draw
Tabs	Yes	No
Explode	Yes	No
Glossary or pronunciations	Yes	Yes
**Educational**		
Library of...	Built-in and custom quizzes, built-in screens, professional courses, recordings	Built-in quizzes, built-in screens, tour of brain and pelvis
Ability to create custom content	Yes: content builder	Yes: tours
Ability to create, save, share dissections	Share directly with others using content builder	Share via email and save to photos
Ability to track student progress	Yes	No
