# Supplementary material for: A case report about anatomy applications for a physical therapy hybrid online curriculum
Source: J Med Libr Assoc. 2020 Apr 1;108(2):295–303. doi: 10.5195/jmla.2020.825 (PMC7069813; doi:10.5195/jmla.2020.825)
Supplement: Appendix B [file jmla-108-295-s002.pdf]

JMLA Journal of the Medical Library Association

Supplemental content to
J Med Libr Assoc. Apr;108(2):dx.doi.org/10.5195/jmla.2020.825
www.jmla.mlanet.org
© Havens, Saulovich, Saric 2020

A case report about anatomy applications for a physical therapy hybrid online curriculum

Kathryn L. Havens; Nicole A. Saulovich; Karin J. Saric

APPENDIX B
Complete Anatomy app student feedback survey questions

1. How often did you use the Complete Anatomy app outside of physical therapy (PT) 514L class time (asynchronous and live session) during fall semester?
a. Every day
b. 2–3 times per week
c. Once a week
d. Less than once a week
e. I never used Complete Anatomy outside of class.

2. How often did you use the Complete Anatomy app outside of PT 554L class time (asynchronous and live session) during spring semester?
a. Every day
b. 2–3 times per week
c. Once a week
d. Less than once a week
e. I never used Complete Anatomy outside of class.

3. How do you like the app?

Not so much	1	2	3	4	5	It was awesome

4. Would you recommend (or have you recommended) the app to someone outside of our program?
a. Yes
b. No

5. Do you think that you will use it in the future, in professional endeavors?
a. Yes
b. No
c. Maybe

6. Is this app useful to help you understand the 3D relationships between structures?
a. Yes
b. No

JMLA Journal of the Medical Library Association

SC

Supplemental content to
J Med Libr Assoc. Apr;108(2):dx.doi.org/10.5195/jmla.2020.825
www.jmla.mlanet.org
© Havens, Saulovich, Saric 2020

7. What aspect of the app do you find most useful?
a. Navigation (getting to the structures you wanted to review)
b. Graphics (resolution of model)
c. Tools (labeling, textboxes, cross-sections, etc.)
d. Other (fill in response)

8. Are there aspects of the app that you do not like?

[Open response]

9. Is there anything else you would like to add regarding the app?

[Open response]
